# WBSA: Web Service for Bisulfite Sequencing Data Analysis

**DOI:** 10.1371/journal.pone.0086707

**Published:** 2014-01-30

**Authors:** Fang Liang, Bixia Tang, Yanqing Wang, Jianfeng Wang, Caixia Yu, Xu Chen, Junwei Zhu, Jiangwei Yan, Wenming Zhao, Rujiao Li

**Affiliations:** Beijing Institute of Genomics, Chinese Academy of Sciences, Beijing, China; UCLA-DOE Institute for Genomics and Proteomics, United States of America

## Abstract

Whole-Genome Bisulfite Sequencing (WGBS) and genome-wide Reduced Representation Bisulfite Sequencing (RRBS) are widely used to study DNA methylation. However, data analysis is complicated, lengthy, and hampered by a lack of seamless analytical pipelines. To address these issues, we developed a convenient, stable, and efficient web service called Web Service for Bisulfite Sequencing Data Analysis (WBSA) to analyze bisulfate sequencing data. WBSA focuses on not only CpG methylation, which is the most common biochemical modification in eukaryotic DNA, but also non-CG methylation, which have been observed in plants, iPS cells, oocytes, neurons and stem cells of human. WBSA comprises three main modules as follows: WGBS data analysis, RRBS data analysis, and differentially methylated region (DMR) identification. The WGBS and RRBS modules execute read mapping, methylation site identification, annotation, and advanced analysis, whereas the DMR module identifies actual DMRs and annotates their correlations to genes. WBSA can be accessed and used without charge either online or local version. WBSA also includes the executables of the Portable Batch System (PBS) and standalone versions that can be downloaded from the website together with the installation instructions. WBSA is available at no charge for academic users at http://wbsa.big.ac.cn.

## Introduction

DNA methylation plays an important role in cell differentiation, X chromosome inactivation, genomic imprinting through regulation of transcription, chromatin structure, chromosome stability, and tumorigenesis [1], [2], [3], [4], [5], [6]. DNA methylation research has been accelerated by the development of next-generation sequencing technology, and it is now the focus of research groups with diverse interests [5], [7], [8], [9], [10], [11], [12]. There are four mainstream sequencing-based methods for DNA methylation profiling: two utilize enrichment of methylated DNA (Methylated DNA Binding Domain sequencing, or MBD-seq [7] and Methylated DNA Immunoprecipitation sequencing, or MeDIP-seq [8], [9]), and the other two utilize bisulfite conversion (MethylC-seq or WGBS [10] and Reduced Representation Bisulfite Sequencing or RRBS [11]). Reacting DNA with bisulfite converts cytosine residues to uracil residues but does not alter 5-methylcytosine residues, which makes it possible to distinguish methylated from unmethylated cytosine residues. Because bisulfite sequencing determines single base changes, its resolution is greater than those methods that utilize DNA enriched in methylated regions [12].

WGBS and RRBS are widely used in biological research [5], [12]. The read alignment of bisulfite sequencing data differs from that generated using non-bisulfite sequencing due to the change of C to T residues. Therefore, alignment tools such as BSMAP [13], BS SEEKER [14], RMAP [15], and Bismark [16] were developed to address this issue. Further, common alignment tools such as BWA [17] and Bowtie [18], [19] also align bisulfite sequencing reads to a reference sequence after the reads and the reference sequences have been converted [10]. Other tools are available for analyzing bisulfite sequencing data, such as CyMATE [20], CpG PatternFinder [21], GBSA [22], COHCAP [23], methylKit [24], and BSmooth [25]. Their applications are limited because their analytical pipelines either require aligned reads as input or only support single-end alignments. Moreover, these tools only identify methylated cytosines or only analyze methylated CpG islands to search for correlations between methylation and gene expression. SAAP-RRBS [26] and RRBS-Analyser [27] integrated BSMAP as an alignment tool and acted as streamlined analysis and annotation pipelines. However, these approaches are designed only for RRBS data with limited annotations. Certain tools identify differentially methylated regions [24], [25], [27], [28], but most do not focus on analysis of non-CGs (Table 1–3).

**Table 1:** Comparison of WBSA's WGBS module with six pipelines.

Functions	WBSA-WGBS	CyMATE	CpG PatternFinder	GBSA	COHCAP	methylKit	BSmooth
Read Quality analysis	Y	N	N	N	N	N	Y
Filter adaptor & low quality	Y	N	N	N	N	N	N
Computation of conversion rate	Y	N	Y	N	N	N	N
Alignment	Y	N	N	N	N	N	Y^a^
Focus on non-CGs	Y	Y	N	Y	N	N	N
Methylation level	Y	Y	Y	Y	Y	Y	Y
Methylation distribution	Y	Y	Y	N	N	N	N
Relationship of methylation and CpG islands	Y	N	N	Y	Y	Y	N
Gene annotation	Y	N	N	Y	N	Y	N
Functional analysis of genes with high or low methylation	Y	N	N	N	N	N	N
Sequence preference	Y	N	N	N	N	N	N
Correlation between methylation and gene expression	Y	N	N	N	Y	N	N
Online version	Y	Y	Y	Y	Y	Y	Y
Standalone version	Y	N	N	N	N	N	N
PBS version	Y	N	N	N	N	N	N

a only support single-end data.

**Table 2:** Comparison of WBSA's RRBS module with three pipelines.

Functions	WBSA-RRBS	SAAP-RRBS	RRBS-analyser	methylKit
Read-quality analysis	Y	Y	Y	N
Filter adaptor & low quality	Y	Y	Y	N
Computation of conversion rate	Y	N	Y	N
Alignment	Y	Y	Y	N
Focus on non-CGs	Y	N	Y	N
Methylation level	Y	Y	Y	Y
Methylation distribution	Y	N	Y	N
Relationship of methylation and CpG islands	Y	N	N	Y
Gene annotation	Y	Y	N	Y
Functional analysis of genes with high or low methylation	Y	N	N	N
Sequence preference	Y	N	N	N
Correlation between methylation and gene expression	Y	N	N	N
Online version	Y	Y	N	Y
Standalone version	Y	N	Y	N
PBS version	Y	N	N	N

**Table 3:** Comparison of WBSA's DMR module with four pipelines.

Functions	WBSA-DMR	RRBS-analyser	BSmooth	methylKit	QDMR
Focus on CGs	Y	Y	Y	Y	Y
Focus on non-CGs	Y	Y	N	N	N
Correlation between DMR and genes	Y	Y	N	Y	Y
The functional analysis of correlative genes	Y	N	N	N	Y
More than one method of DMR identification	Y	N	N	N	N
Online version	Y	Y	Y	Y	Y
Standalone version	Y	N	N	N	N
PBS version	Y	N	N	N	N

We describe here WBSA, which provides a user-friendly and novel web service for analyzing bisulfite sequencing data. WBSA focuses on the analysis of CpG as well as CHG and CHH (H = A, T or C), and therefore aids DNA methylation research related to animals or plants. Researchers can use WBSA to perform comprehensive analyses of WGBS or RRBS data and can identify DMRs in different contexts. Moreover, WBSA is efficient and fast. For example, the overall process starting from the analysis of the quality of reads to annotating methylation only takes 5.5 hours for 2.8 GB (15.6M reads) sample data with WGBS. WBSA comprises six modules: Home, WGBS, RRBS, DMR, Documents, and Downloads and provides the executables for downloads and local installation.

## Methods

### 1) Analysis and annotation of bisulfite sequencing data

**Figure 1:**
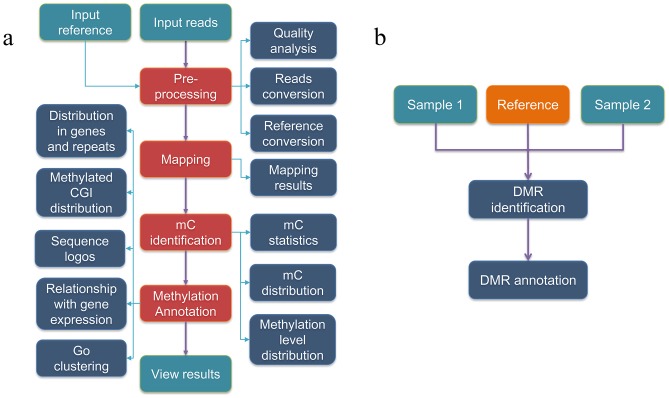
Flowchart of data analysis. a. Flowchart of data analysis for WGBS and RRBS. WGBS and RRBS include four parts as follows: pre-processing of reads and the reference sequence, mapping to the reference genome, mC identification, and methylation annotation. The sequencing reads, reference sequences, and the lambda sequence should be used as input data, and all the results can be previewed and downloaded. b. Flowchart of DMR identification. The DMR analysis module includes DMR identification and annotation.

WBSA is a web service for analyzing bisulfite sequencing data and uses Perl and C to compile all program scripts. WBSA comprises three main (WGBS, RRBS and DMR) and three basic (Home, Documents and Downloads) modules. The main modules analyze and annotate the bisulfite sequencing data. The flowcharts shown in Figure 1 depict the process of data analysis. Detailed methods are provided below.

Data uploading and analyzing read quality: WBSA provides two modes for uploading sequencing data: HTTP (for files <2 GB) and FTP (no size limit). We integrated FastQC software (http://www.bioinformatics.babraham.ac.uk/projects/fastqc/) to analyze the quality of bisulfite sequencing data. WBSA generates figures and tables that show the results of the analysis of quality distribution, nucleotide distribution, GC content distribution, and overrepresented sequences as well as other information. The user can filter adaptor sequences and low quality bases from two ends of a sequence if the base quality value is less than the value of the threshold (optional parameter) for WGBS and RRBS data. If the read length is less than the minimum read length (predefined value) after filtering bases, the reads will be discarded.

Sequence alignments: For bisulfite sequencing reads, cytosines in T-rich reads are replaced with thymines, while guanines in A-rich read are replaced with adenines. The position of the replaced cytosines or guanines will be marked if the quality value is larger than Q (a predefined value). WBSA prepares the reference sequence and simultaneously converts it twice as follows: (1) cytosines are replaced with thymines, and (2) guanines are replaced with adenines. BWA [17] is used to align processed reads according to the converted reference sequence. The default mapping parameters can be changed by the user. If an unmethylated DNA sequence Lambda named “chrLam” is used and uploaded, WBSA can integrate the Lambda sequence within the reference sequence. The Lambda genome is included in the reference sequence as an extra chromosome so that reads originating from the unmethylated control DNA can be aligned. The sodium bisulfite non-conversion rate is calculated as the percentage of cytosines sequenced at cytosine reference positions in the Lambda genome. WBSA can process single-end and paired-end data for WGBS, but only processes single-end data for RRBS, because the restriction endonuclease digestion fragments are likely to be shorter (40–220 bp). Therefore, single-end sequencing is more practical to perform than paired-end sequencing. WBSA discards four types of reads that map to the reference as follows: (1) reads mapped to multiple positions; (2) reads mapped to the wrong strands (T-rich reads mapped to Crick-strand Cs converted to Ts or to Watson-strand Gs converted to ‘A’s, A-rich reads mapped to Watson-strand Cs converted to Ts or to Crick-strand Gs converted to ‘A’s). WBSA only supports analysis of methylC-seq data, which is strand-specific; (3) T-rich reads where a C maps to T in the reference sequence, or A-rich reads where a G maps to an A in the reference sequence; and (4) duplicated reads generated by the use of PCR (optional parameter).

Identification of methylation sites: For each reference cytosine, WBSA uses the binomial distribution B(n, *p*) to identify the methylation site, using a 0.01 false discovery rate (FDR) corrected P-value [10], where the probability *p* in the binomial distribution B(n, *p*) is estimated from the number of cytosines sequenced in reference sequence cytosine positions in the unmethylated Lambda sequence (referred to as the error rate: non-conversion plus sequencing error frequency) if the Lambda sequence is uploaded by the user; otherwise, the probability *p* must be provided by the user. For each reference cytosine, the trial number (n) is the read depth, and the cytosine is noted as methylated if the number of sequenced cytosines (m) follows the following formula as below:
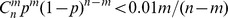
Further, the RRBS module eliminates the impact on mC identification because of double strand DNA repair and conversion into blunt ends at the terminus of a sequence.

Annotation by WGBS and RRBS: WBSA provides a wide variety of annotations and analyses for WGBS and RRBS. WBSA first evaluates the abundance of methylated cytosines in the genome and shows the distribution of methylation in different regions (upstream, first exon, first intron, internal exons, internal introns, last exon, downstream) of genes and in repeats. WBSA next performs a statistical analysis of the number and percentage of methylated CpG islands in different functional genic regions (promoter, gene body, downstream, and intergenic). A methylated CpG island is defined as a sequence of 200-plus base pairs with a G+C content of greater than 50%, the observed/expected C frequency of greater than 0.6 and a methylation level of greater than 70%. The third is the functional clustering analysis of genes with high and low levels of methylation. Functional gene clustering is implemented using three steps: (1) methylation level of each gene is counted; (2) genes with high (>70%) and low (<30%) levels of methylation are annotated and functionally classified according to Gene Ontology (GO) terms, respectively; (3) the numbers of genes with the high and low levels of methylation are counted, and histograms are generated (horizontal axis and vertical axes represent the functional class and gene number, respectively). Fourth, a red graph shows the distribution of methylation levels in transposable elements (TE). Fifth, the sequence preference for mCG, mCHG, and mCHH are analyzed using WEBLOGO software [29]. Sixth, the correlation between gene expression and methylation levels is analyzed, and this analysis consists of four steps as follows: (1) uploaded genes are sorted according to the expression values; (2) sorted genes are divided equally into five groups, such that the first group contains genes with the lowest expression values; (3) each gene body or promoter region is divided equally into 20 bins, and the average relative methylation level of each bin for genes in every group is calculated; (4) two-dimensional curves are generated (horizontal axis, gene body or promoter region; vertical axis, average relative methylation level), showing the relative levels of mCG, mCHG, and mCHH contexts in the promoter regions and gene bodies for WGBS and the CG context for the RRBS promoter regions.

Identification of differentially methylated regions: WBSA includes an independent module for DMR identification (Figure 1b) and provides the static window and dynamic window methods. The static window method is used to identify DMRs in strings of CN, CG, and CH (N = A, T, C or G, H = A, C or T). This approach fixes the window length and the number of adjacent windows. The Wilcoxon test is used if both samples have sufficient coverage in these windows and the methylation level of one sample is greater, at least 0.2 (delta methylation level), than that of the other. The test window moves one mC for each step. The p-value, minimum sequence coverage rate and delta methylation level can be adjusted according to user's expectations. Whether using FDR correction is determined by users. The dynamic window method is used to identify DMRs in strings of CN and CG. The Wilcoxon test is used in a window with fixed numbers of CNs or CGs if the coverage of both samples is sufficient and the methylation level of one sample is greater, at least 0.2 (delta methylation level), than that of the other. First, the window moves towards the 3′-direction one step-size at a time and repeats the Wilcoxon test until the *p*-value is not significant or until the end of the sequence is reached. The same process is repeated in the original fixed window in the 5′-direction. The window size, step size, coverage, delta methylation level and *p*-value can be adjusted in accordance with different expectations.

There are three sample data analysis pipelines showing all corresponding analyses on the WGBS, RRBS, and DMR pages, and users can access the corresponding links to analyze these sample datasets or check the results using WBSA. A detailed description of how to use WBSA is shown on the Documents page. Links for all the software related to the service and instruction manuals, which supports the PBS and a standalone versions, are provided on the Downloads page.

### 2) System architecture

**Figure 2:**
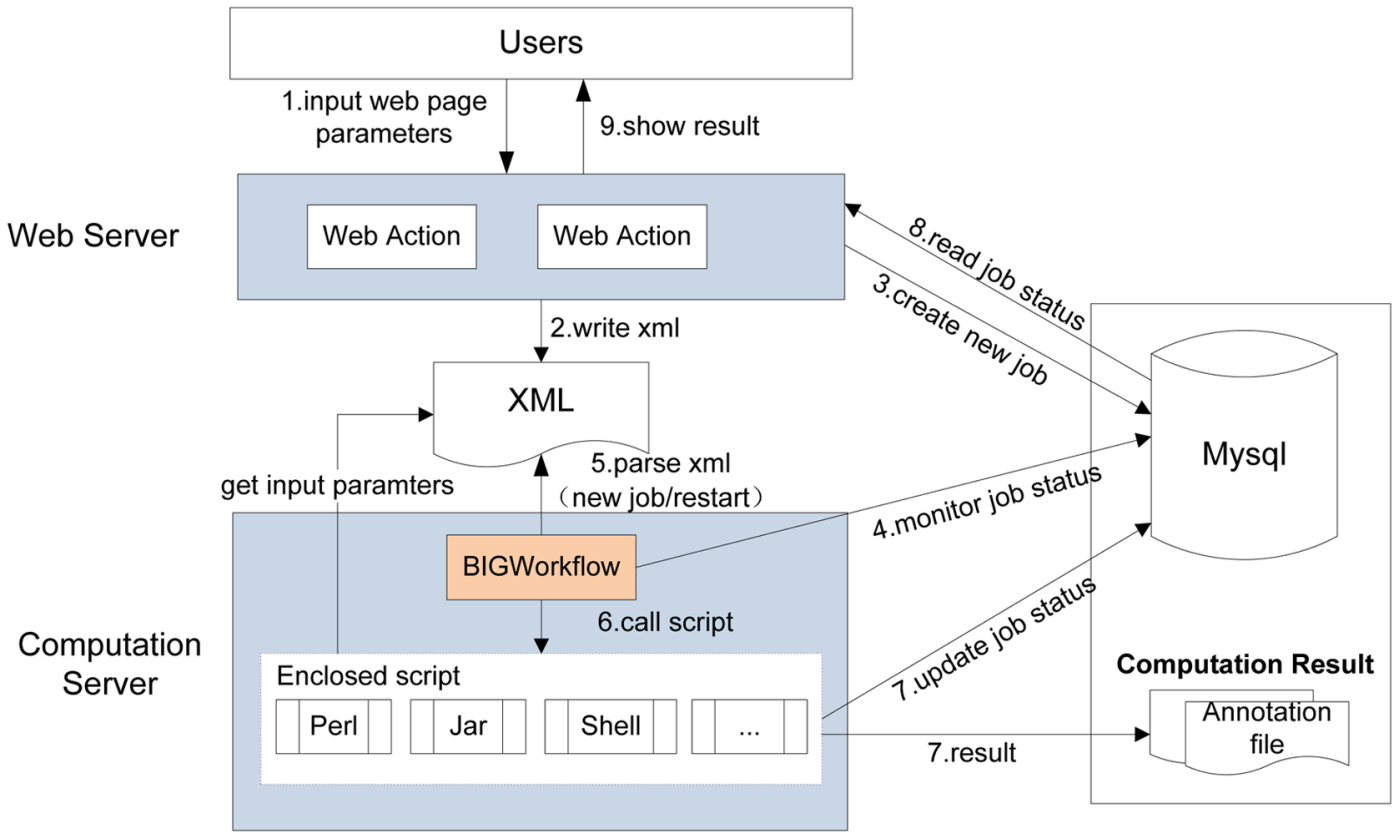
WBSA system architecture and workflow. When the user chooses one analysis module from the web page such as WGBS, the user must input several parameters according to the instructions provided. The web process, which was developed using a Struts and Spring framework, will then proceed with the user's request and generate an XML file to store the parameters provided by the user. At the same time, it will insert a record into the database to identify the new job. The workflow monitor process BIG Workflow will continually monitor the job's status from the database and will activate the data processing procedure if a new job is found. When the user previews the result on the web page, the web process will indicate the status of the job and show the appropriate results to the user.

WBSA consists of a web application and computational modules, which we take as front-end and back-end programs, respectively [30]. The front-end program was developed using Java Server Page (JSP) technology, which is used to process the submitted task request and shows the results. Three popular frameworks, Struts (http://struts.apache.org), Spring (http://www.springsource.org), and MyBatis (http://mybatis.github.io), are simultaneously used to enhance the flexibility and extendibility of the web application. The Mysql database is used to store information, which will be used by the front-end and back-end programs. The back-end program was mainly developed using Perl to process the data. A Java package encloses all the Perl scripts, monitors task status, and executes the Perl script. An XML file is used to exchange information between front-end and back-end programs. The system architecture and workflow are shown in Figure 2.

The executable package includes PBS and standalone versions, which are available for download from our website and can be installed locally on a Linux-64 system. The executables include a Jar package and several configuration files. Users only need to write a configuration file and then start the Jar package to activate the task. Some essential tools described on the download page require installation to enable the system to run.

### 3) Simulation of bisulfite sequencing data

To simulate WGBS data, we first used DWGSIM software (version 0.1.7) (https://github.com/nh13/DWGSIM) to simulate NGS paired-end 80-bp reads with a 0.5% base-error rate on a whole-genome scale for two model organisms (zebrafish and rice). And the simulated data includes 5% random reads. The simulation parameters are -e 0.005 (base-error rate of the first read sequences) -E 0.005 (base-error rate of the second read sequences) -C 10 (10-fold coverage) -1 80 (length of the first read sequences) -2 80 (length of the second read sequences) -y 0.05 (5% random reads). In the second step, Cs are randomly converted to Ts for the first-read sequences of paired-end reads and Gs to ‘A’s for the second-read sequences of paired-end reads. The numbers of simulated reads include 89,278,622 and 24,677,386 pairs, respectively, and represent 10-fold coverage of the zebrafish and rice genomes. The numbers of random DNA sequences were 4,492,050 and 1,235,216 pairs, respectively. We trimmed 10 and 20 bases from the ends of simulated reads and generated 70 and 60 bp long reads.

To simulate RRBS data, first we scanned either the human (hg19) or mouse (mm9) genome and marked the positions of CCGGs for the Watson and Crick strands, and the distance between adjacent CCGGs should be ≥40 bp and ≤220 bp. Then we extracted at random 36-bp sequences that start with CGG (starting with CCGG and removing the first C). Next, we introduced randomly 0.5% incorrect bases into these 36-bp fragments and then imported 5% random DNA sequences. In the final step, we converted at random Cs to Ts in each read. The total numbers of simulated reads of human and mouse were 17,087,814 and 7,463,343, and the numbers of random DNA sequences were 854,403 and 373,182 reads, respectively.

## Results and Discussion

### 1) Evaluation of the mapping efficiency and accuracy of WBSA

Mapping reads to a reference genome is an important step for the analysis of bisulfite sequencing. We therefore compared WBSA with the two most popular mapping software packages, Bismark and BSMAP. The comparison includes the following variables: sequencing types (paired-end and single-end), read length (80, 70, 60, and 36 bp), data types (simulated data and actual data), and library types (WGBS and RRBS data). We simulated paired-end reads with different lengths of zebrafish and rice genomes for WGBS and single-end reads of human and mouse genomes for RRBS (simulation methods are described in the Methods section). We used three methods (WBSA, BSMAP and Bismark) to align simulated and actual sequencing reads to their corresponding genomes. The results show that WBSA performed as effectively as BSMAP and Bismark. In contrast, WBSA mapping was more accurate and faster. The detailed results are presented in Table 4–6.

**Table 4:** Comparison of mapping times and accuracies among WBSA, BSMAP, and Bismark for simulated WGBS data.

Read length (bp)	Species	Software	Alignment Parameters	Mapping Time (hours)	RAM (Gb)	Mapped Reads		Correctly Mapped Reads		False Positive		False Negative	
						Num. (pairs)	%	Num. (pairs)	%	Num. (pairs)	%	Num. (pairs)	%
80	Zebrafish	Bismark (v0.8.1)	-q –phred33-quals -n 3 -l 16	47.80	∼5.5	78,801,150	88.26	77,891,346	87.25	0	0	5,985,422	6.70
		BSMAP (v2.74)	-s 16 -v 3 -p 1 -r 1 -R -u	7.60	∼4.3	84,439,556	94.58	70,308,940	78.75	0	0	347,016	0.39
		WBSA	-n 3 -l 16 -k 3	24.07	∼4.3	84,776,394	94.96	80,698,421	90.39	0	0	10,178	0.01
	Rice	Bismark (v0.8.1)	-q –phred33-quals -n 3 -l 16	12.57	∼1.5	21570946	87.41	21266096	86.18	0	0	1871224	7.58
		BSMAP (v2.74)	-s 16 -v 3 -p 1 -r 1 -R -u	1.18	∼1.7	23416611	94.89	20235903	82.00	0	0	25559	0.10
		WBSA	-n 3 -l 16 -k 3	5.47	∼1.2	23442162	94.99	23289124	94.37	0	0	8	0
70	Zebrafish	Bismark (v0.8.1)	-q –phred33-quals -n 3 -l 16	40.37	∼4.3	78160397	87.55	77067467	86.32	0	0	6626175	7.42
		BSMAP (v2.74)	-s 16 -v 3 -p 1 -r 1 -R -u	11.45	∼4.3	84383101	94.52	72790003	81.53	0	0	403471	0.45
		WBSA	-n 3 -l 16 -k 3	25.45	∼4.3	84786567	94.97	84697662	94.87	0	0	5	0
	Rice	Bismark (v0.8.1)	-q –phred33-quals -n 3 -l 16	10.72	∼1.2	21390366	86.68	21034061	85.24	0	0	2051804	8.31
		BSMAP (v2.74)	-s 16 -v 3 -p 1 -r 1 -R -u	1.03	∼1.8	23422665	94.92	19760196	80.07	0	0	19505	0.08
		WBSA	-n 3 -l 16 -k 3	4.92	∼1.2	23442166	94.99	23121395	93.69	0	0	4	0
60	Zebrafish	Bismark (v0.8.1)	-q –phred33-quals -n 2 -l 14	39.77	∼5.1	77325014	86.61	76000508	85.13	0	0	7461558	8.36
		BSMAP (v2.74)	-s 14 -v 2 -p 1 -r 1 -R -u	8.05	∼4.3	84242377	94.36	70017299	78.43	0	0	544228	0.61
		WBSA	-n 2 -l 14 -k 2	15.93	∼4.3	84786571	94.97	84068061	94.16	0	0	1	0
	Rice	Bismark (v0.8.1)	-q –phred33-quals -n 2 -l 14	9.53	∼1.5	21158772	85.74	20741988	84.05	0	0	2283398	9.25
		BSMAP (v2.74)	-s 14 -v 2 -p 1 -r 1 -R -u	0.77	∼1.7	23412528	94.87	19161765	77.65	0	0	29642	0.12
		WBSA	-n 2 -l 14 -k 2	3.94	∼1.1	23442168	94.99	22910455	92.84	0	0	2	0

**Table 5:** Comparison of mapping times and accuracies between WBSA, BSMAP, and Bismark for simulated RRBS data.

Species	Software	Alignment Parameters	Mapping Time (hours)	RAM (Gb)	Mapped Reads		Correctly Mapped Reads		False Positive		False Negative	
					Num.	%	Num.	%	Num.	%	Num.	%
Human	Bismark (v0.8.1)	-q –phred33-quals -n 2 -l 14	5.54	∼10.5	10930929	67.63	10849359	67.13	795	0	5303277	31.04
	BSMAP (v2.74)	-s 14 -v 2 -p 1 -r 1 -R -u	1.22	∼7.5	16161772	94.58	12489088	73.09	23	0	71662	0.42
	WBSA	-n 2 -l 14 -k 2	1.42	∼6.3	16228389	94.97	12302379	72.00	264	0	5286	0.03
Mouse	Bismark (v0.8.1)	-q –phred33-quals -n 2 -l 14	1.52	∼7.1	5099599	68.3	5065633	67.87	206	0.06	1990768	26.67
	BSMAP (v2.74)	-s 14 -v 2 -p 1 -r 1 -R -u	0.28	∼6.8	7054102	94.52	5603328	75.08	5	0	36064	0.48
	WBSA	-n 2 -l 14 -k 2	0.63	∼6.1	7087675	94.97	5594941	74.97	51	0.01	2537	0.03

**Table 6:** Comparison of mapping times and accuracies between WBSA, BSMAP, and Bismark for actual bisulfite sequencing data.

Data type	Species	Software	Alignment Parameters	Mapping Time (hours)	RAM (Gb)	Mapped Reads		Uniquely Mapped Reads	
						Num.	%	Num.	%
WGBS	Human	Bismark(v0.8.1)	-q –phred33-quals -n 3 -l 16	303.9	∼10.6	166849837	37.33	153969814	34.45
		BSMAP(v2.74)	-s 16 -v 3 -p 1 -r 1 -R -u	42.73	∼8.0	238134054	53.28	220938793	49.43
		WBSA	-n 3 -l 16 -k 3	113.20	∼9.2	240834825	53.88	222198832	49.71
RRBS	Mouse	Bismark(v0.8.1)	-q –phred33-quals -n 2 -l 14	22.65	∼9.1	17609963	85.30	12893165	62.45
		BSMAP(v2.74)	-s 14 -v 2 -p 1 -r 1 -R -u	3.93	∼6.8	12489362	60.50	9137791	44.26
		WBSA	-n 2 -l 14 -k 2	5.14	∼8.0	13250668	64.19	9533829	46.18

For mapping simulated WGBS paired-end data with different lengths, the three mapping methods had a false-positive rate of zero. BSMAP ran the fastest, followed by WBSA, and Bismark. However, WBSA produced the highest mapped rates, the correctly mapped rates, and the lowest false negative rates. The correctly mapped rate is the ratio of the correctly mapped simulated reads to the total simulated reads, and the false negative rate is the ratio of the simulated unmapped, nonrandom reads to total simulated reads. There was little difference in memory use among the methods (Table 4).

For mapping simulated RRBS single-end data, memory use, mapping times, mapped rates, correctly mapped rates, false negative rates, false positive rates of the WBSA and BSMAP methods were similar. Each out-performed Bismark (Table 5).

We downloaded the actual WGBS data for human (SRX006782, 447M reads) and actual RRBS data for mouse (SRR001697, 21M reads) from the website of the United States National Center for Biotechnology Information (NCBI) to compare the mapped rates and uniquely mapped rates of WBSA with BSMAP and Bismark. The results show that mapped rates or uniquely mapped rates of WBSA were superior to that of BSMAP. The uniquely mapped rates of Bismark were the highest for the mouse dataset, but both rates were not comparable with those of WBSA or BSMAP for the human dataset. The mapping time and memory use for WBSA fell between those of BSMAP and Bismark (Table 6). Considering all of the above results, we conclude that the WBSA mapping method was more accurate and efficient than the other two methods.

### 2) Evaluation of the accuracy of WBSA analysis

**Figure 3:**
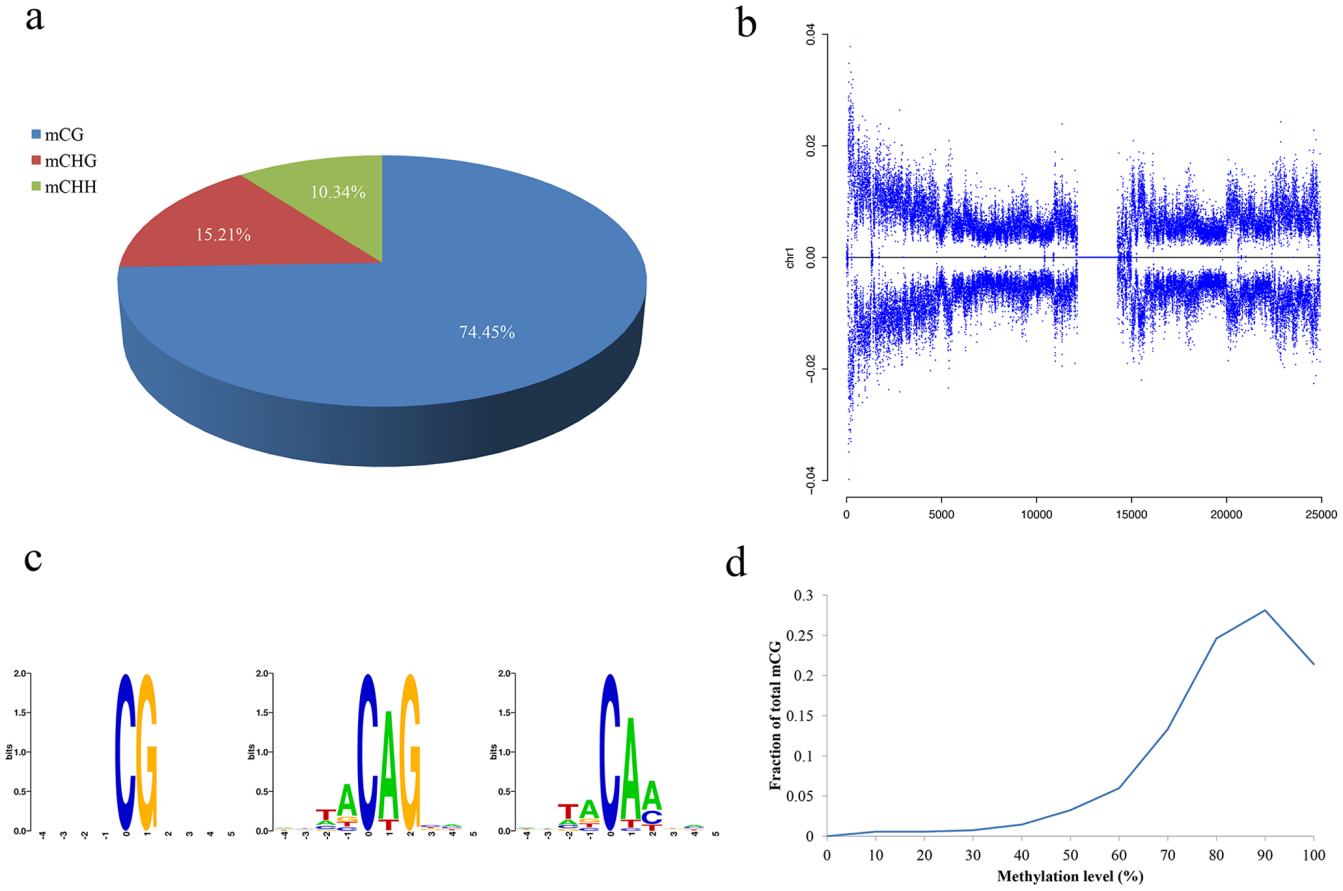
The performance of WBSA compared with a published study. a. The percentage of methylcytosine identified in each sequence context. b. The methylcytosine density in Chr1. Each dot indicates the methylation density in a 10-kb window. c. Logo plots of sequences proximal to sites of DNA methylation in each sequence context. Logos are presented for all methylcytosines. Three or four bases flanking each methylcytosine context were analyzed to show the local sequence preference. d. Distribution of the methylation level in the CG context. The vertical axis indicates the fraction of methylated CGs for a corresponding methylation level (horizontal-axis) where the methylation level is defined as the mCG∶CG ratio at each reference cytosine in the CG context (at least 10× coverage is required).

To estimate the accuracies of the identification of methylation sites and the advanced analysis results generated by WBSA, we downloaded the published embryonic stem cell dataset from the NCBI website (SRA accessions SRX006239–41, 1.12 G reads). The data are derived from the report of Lister et al. [10], who presented the first genome-wide, single-base resolution maps of methylated cytosines in a mammalian genome from human embryonic stem cells and fetal fibroblasts. The entire analysis took about approximately five days, reads of three libraries were pre-processed as the same time first, then they were mapped simultaneously to the reference sequence, finally the combined data were further analyzed sequentially. We found that our annotation results were consistent with those of Lister et al. [10]. For example, the bisulfite conversion rate for WBSA and Lister et al. were 99.7% and 99.6%, respectively. This small difference may be accounted for by more extensive filtering by WBSA. For instance, post-analysis by WBSA filtered out the following: T-rich reads that mapped Cs to Ts in the reference genome; A-rich reads that mapped Gs to ‘A’s in the reference genome; T-rich reads that mapped to Crick strands of Cs that were converted to Ts or Watson strand Gs that were converted to ‘A’s, and A-rich reads that mapped to Watson strand Cs converted to Ts, or Crick strand Gs converted to ‘A’s. For the identified mCs, non-CGs accounted for approximately 25% of all mCs, and the number of mCHHs was the lowest, which is consistent with the published data (Figure 3a). We also observed that the distribution of mC for all chromosomes was almost the same shape as that published by Lister et al. (Figure 3b, Figure S1). Further, we did not detect local sequence enrichment for mCGs, but did find a preference for TA dinucleotides upstream of non-CG methylated regions. The base following a non-CG methylcytosine was most commonly an A, and a T was also observed frequently. This is the same as the preference in the paper (Figure 3c). The distribution of methylation levels shows that most of the CGs is highly methylated, consistent with results of Lister at al. (Figure 3d).

## Conclusions

WBSA is an interactive web-based service that was designed for researchers who may not necessarily be familiar with post-analysis of bisulfite sequencing data or for those lacking local computing resources. WBSA is a free, accurate, comprehensive, and user-friendly tool for analyzing bisulfite sequencing data that integrates read-quality analysis, read preprocessing, read mapping, mC identification, and annotation analysis. WBSA focuses on CG and non-CG methylation, and can be applied to DNA methylation research for animal and plant genomes. WBSA is a highly automated package that can be run in a local cluster environment or on a standalone server.

## Supporting Information

Figure S1 **The methylcytosine density in all chromosomes.**
(TIF)
