# Supplementary figures and images for: WBSA: Web Service for Bisulfite Sequencing Data Analysis
Source: PLoS One. 2014 Jan 30;9(1):e86707. doi: 10.1371/journal.pone.0086707 (PMC3907392; doi:10.1371/journal.pone.0086707)

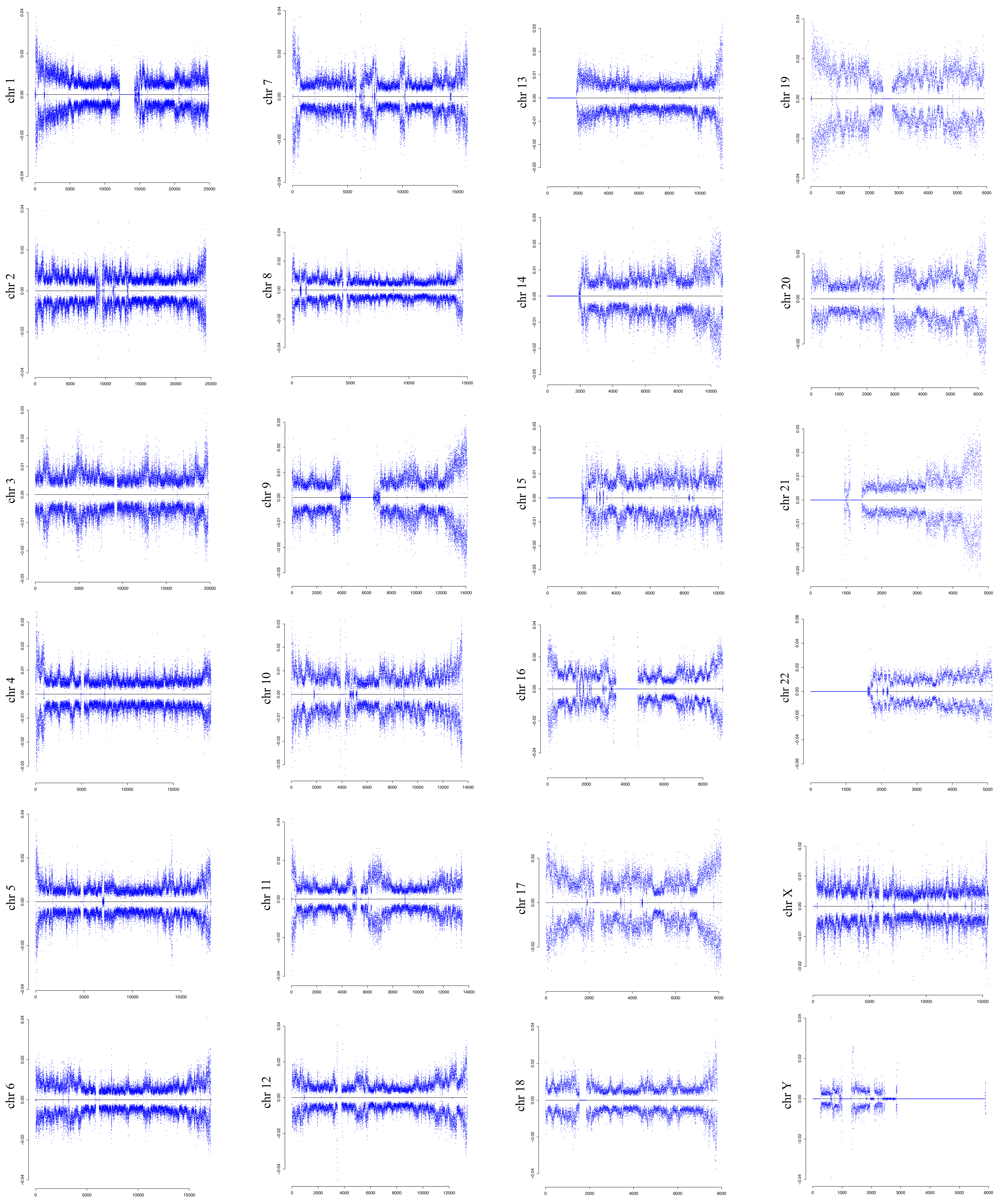

Supplement: Figure S1 — The methylcytosine density in all chromosomes. (TIF) [file pone.0086707.s001.tif]
